# Emotional Flow in Vaccine Communication: Psychophysiological and Persuasive Responses to Preventive Health Messages

**DOI:** 10.3390/bs16091634

**Published:** 2026-09-13

**Authors:** Yen-I Lee, Isabelle Martinez, Paul Bolls

**Affiliations:** 1 Murrow College of Communication, Washington State University, Pullman, WA 99164, USA; isabelle.martinez@wsu.edu; 2 Jake Jabs College of Business and Entrepreneurship, Montana State University, Bozeman, MT 59717, USA; paul.bolls@montana.edu

**Keywords:** emotional flow, narratives, vaccination, preventive health campaigns, psychophysiological approach

## Abstract

Introduction: This study unfolds how embodied emotional processes vary across time and impact conscious attitudinal toward COVID-19-related policy when individuals experience narratives with different emotional features about vaccination for public health crisis prevention. Methods: A media psychophysiology lab experiment was conducted in which college students (N = 86) were exposed to videos that varied in emotional flow. Psychophysiological indicators of arousal (skin conductance), cognitive resource allocation (heart rate), and negative emotional response (corrugator facial EMG) were recorded during exposure to stimulus videos, and self-report measures of attitudes were obtained after each video. Results: Emotional flow impacted the cognitive resources allocated to encoding and moment-by-moment arousal and emotional responses. Health messages with emotional flow features elicited a more skeptical emotional response, whereas those without emotional flow features led to stronger feelings of hope and more positive attitudes toward university public health prevention policy about vaccination. Conclusions: Narrative vaccine campaigns with emotional flow increased psychophysiological engagement but did not improve persuasive outcomes in this experimental context because messages containing emotional shifts elicited greater skepticism and less favorable policy attitudes than consistently positive messages about the COVID-19 vaccine.

## 1. Introduction

Evidence-based communication strategies that incorporate emotional content may help address these barriers by capturing people’s attention and increasing engagement with vaccination messages (Chou & Budenz, 2020). For instance, previous research has shown that celebrity endorsement combined with hope appeals could influence COVID-19 vaccination intentions (Lee et al., 2024), while entertainment-based vaccine videos featuring participants’ favorite music could elicit positive emotions and motivate behavioral change among vaccine-hesitant individuals (Duong et al., 2024). These findings suggest that emotional content can play an important role in attracting and sustaining individuals’ engagement with vaccination messages.

However, emotional engagement with a persuasive message does not necessarily translate into favorable persuasive outcomes. Emotional appeals can increase message relevance and engagement while also intensifying defensive processing, skepticism, or psychological reactance when individuals perceive the message as threatening, coercive, or inconsistent with their beliefs (Clayton, 2022; Nabi, 2010). An important question is not simply whether emotional messages increase engagement, but how dynamic emotional experiences during message exposure shape processing and whether heightened engagement subsequently translates into persuasion.

Emotional flow provides a useful framework for addressing this question because it conceptualizes emotional responses as dynamic rather than static. Within persuasive health communication research, emotional flow has emerged as an important framework for understanding how emotional experiences dynamically change throughout a message rather than remaining emotionally static (Nabi, 2015; Nabi & Green, 2015). For example, a message may move individuals from negative emotional states associated with threat or loss toward positive states associated with hope or anticipated benefits. Unlike research examining the effects of a single discrete emotion, an emotional flow perspective focuses on how changes in emotional experience unfold over time and how these changes may influence subsequent cognitive, affective, and evaluative responses. Importantly, emotional flow should not necessarily be understood as an inherently persuasive feature of a message. Although emotional shifts may increase attention, transportation, or emotional engagement (Alam & So, 2020; Hundal et al., 2024; Winkler et al., 2023), heightened engagement may facilitate more intensive processing without determining the direction of subsequent evaluations. Thus, emotional flow may function as an amplifier of message processing, with its consequences for persuasion depending on how audiences interpret and evaluate the message.

Emotional appeals may increase message relevance and motivate protective health behaviors, yet they may also intensify defensive processing, skepticism, or psychological reactance when audiences perceive messages as threatening or coercive (Clayton, 2022; Nabi, 2010). Within persuasive health communication research, emotional flow has emerged as an important framework for understanding how emotional experiences dynamically change throughout a message rather than remaining emotionally static (Nabi, 2015; Nabi & Green, 2015). Emotional flow refers to variation in emotional valence and arousal over time, such as shifts from fear to hope, from loss to gain, or from uncertainty to reassurance. Previous research suggests that emotional shifts may increase narrative transportation, affective forecasting, and persuasive engagement in health communication contexts (Alam & So, 2020; Hundal et al., 2024; Winkler et al., 2023). Research examining fear-hope sequencing in vaccination messaging has further suggested that pairing threatening information with hopeful efficacy-based outcomes may influence vaccination-related attitudes and support for preventive health behaviors (Lu & Yuan, 2023; Nabi & Myrick, 2019). Emotional flow refers to a dynamic movement of emotional experience across a message with different features. Unlike examining the effects of a single discrete emotion, the perspective of emotional flow focuses on how changes in emotional states unfold over time and how these changes may shape subsequent cognitive and affective responses to a message. At the same time, emotional flow may not universally strengthen persuasion. Emotional shifts that intensify emotional arousal may also heighten uncertainty, skepticism, or resistance among vaccine-hesitant audiences. Thus, emotional flow represents a potentially powerful but theoretically complex feature of vaccine communication.

Despite extensive research on vaccine attitudes and persuasive health messaging, psychophysiological approaches remain rare within vaccine communication research. A recent systematic review of randomized controlled trials on vaccine communication concluded that many persuasive message features produce limited effects on self-reported attitudes and behavioral intentions (Xia & Nan, 2024). These findings suggest that vaccine communication research may benefit from moving beyond traditional self-report measures to better understand the underlying cognitive and emotional processes occurring during message exposure. Persuasive health messages are processed through embodied cognitive and motivational systems that continuously shape attention, emotional arousal, and emotional response (A. Lang, 2006, 2009). The Media Psychophysiology Paradigm provides a framework for examining these real-time embodied processes by integrating physiological measurement with communication theory to understand how audiences cognitively and emotionally respond to media content (Bolls et al., 2019; Lee et al., 2023). Recent psychophysiological health communication research suggests that physiological responses can reveal implicit attentional and emotional processes not fully reflected in self-report outcomes alone (Acconito et al., 2025). This gap is important within vaccine communication research because vaccine hesitancy is strongly associated with emotional uncertainty, defensive processing, and distrust. This gap is also important for emotional flow because its defining characteristic is the temporal movement of emotional experience across messages. Psychophysiological measures provide a complementary approach by capturing changes in physiological responding continuously during exposure, thereby offering insight into cognitive resource allocation, motivational arousal, and emotional responding that may not be fully reflected in retrospective self-reports. Understanding physiological responses to vaccine messages may therefore help explain why some persuasive strategies successfully attract attention while simultaneously producing unintended skepticism or resistance.

Grounded in emotional flow (Nabi & Green, 2015), the media psychophysiology paradigm (Bolls et al., 2019), and the Limited Capacity Model of Motivated Mediated Message Processing (LC4MP; A. Lang, 2006), this study examines whether and how emotional shifts influence both real-time psychophysiological processing and subsequent conscious evaluation of vaccination-related messages. LC4MP suggests that cognitive resources, emotional arousal, and motivational systems interact dynamically during media exposure, shaping how individuals attend to, encode, and emotionally respond to persuasive content (A. Lang, 2006, 2009). From this perspective, emotional shifts may alter the allocation of cognitive resources and motivational activation as individuals encounter different content features of a message. Importantly, increased allocation of processing resources does not necessarily imply greater acceptance of the message. Rather, heightened engagement may increase the extent to which individuals process and respond to message content, while the evaluation outcome may depend on how the content is interpreted.

## 2. Literature Review

### 2.1. Emotional Flow and Real-Time Psychophysiological Message Processing

Emotional flow is conceptualized as variation in emotional experience during exposure to a persuasive message, which consists of emotional shifts (Nabi, 2015; Nabi & Green, 2015). This conceptualization builds on dimensional approaches to emotion, which organize emotional experience primarily along valence and arousal dimensions (Bradley & Lang, 2007; Russell, 2003), while also recognizing that changes in specific discrete emotions can occur as message content unfolds (Izard, 2007; Lazarus, 1991). From the emotional flow perspective, a single message may generate successive changes in emotional valence, arousal, or discrete affective states. The theoretical significance of emotional flow lies not simply in the presence of emotion, but in the transition between emotional states over time.

Research focused on the persuasive effects of emotional flow, such as emotional shifts within a message are in the early stages. Some experiments provide evidence that emotional shifts in a message may lead to increased transportation—an experience related to stronger narrative persuasion (Alam & So, 2020; Winkler et al., 2023). However, Alam and So’s (2020) study had the emotional flow manipulation issue that was confounded with message length, so it was difficult to distinguish whether emotional shifts or differences in message exposure led to heightened engagement. Moreover, emotional flow was operationalized primarily as a single shift between positive and negative valence. To address measurement limitations, Winkler et al.’s (2023) study developed a self-probed emotional retrospection approach to capture the number and intensity of emotional shifts experienced throughout narratives; however, the approach they developed still relies on subjective reports of emotional experience rather than continuous psychophysiological indicators of message processing. Lu and Yuan (2023) recently found evidence that an emotional content shift from fear to hope in a video promoting MMR vaccination resulted in greater intentions to engage in activism (e.g., supporting mandatory vaccination) but only among individuals who perceive the issue to be highly relevant. Although their study demonstrated the influence of emotional flow manipulated by sequencing fear and hope on persuasive outcomes and assessed fear and hope at multiple points during message exposure, relying on self-reported emotional experiences limited the ability to capture uninterrupted and moment-to-moment processing of emotional flow. Hundal et al. (2024) found that patient testimonials containing an emotional shift in the intensity of fear improve affective forecasting—the ability to manage emotions evoked by a health risk—resulting in better health decision-making. Although they manipulated emotional shifts as the intensity of a single negative emotion (e.g., fear), their study remains insufficiently understood regarding the transition between discrete emotional states and the underlying process through which emotional flow impacts persuasion.

Other researchers have concluded that emotional content shifts—specifically mixed framing consisting of both gain and loss frames—might be more effective at increasing health behavior intentions but not necessarily actual behavior (Ort et al., 2023). Although they operationalized emotional flow through message structure and assessed it using post-exposure reports of arousal and positive emotional experience, it remained unknown whether participants actually experienced dynamic emotional changes as the message unfolded. Ort et al. (2023) suggested using real-time and psychophysiological measures to capture the emotional changes during message exposure. Taken together, these findings suggest that emotional shifts can influence how individuals experience and respond to health messages. The results from these studies also indicate that emotional flow may influence the intensity or quality of message processing without necessarily determining the direction of persuasive outcomes. Also, more research is needed to investigate the real-time psychological processes that incorporate emotional flow during message exposure.

Researchers who study the persuasive effects of emotional flow within health communication have exclusively investigated effects on self-reported outcome measures related to persuasion, leaving the real -time processing mechanisms underlying emotional shifts relatively unexplored. This limitation is consequential because retrospective self-report cannot fully capture the temporal dynamics that define emotional flow. The limited psychophysiological evidence from related research provides initial support for examining physiological responses as complementary indicators of processing during emotional shifts. For example, the most related experiment by Clayton et al. (2021) investigated the experience of mixed affect—a form of emotional flow—in what has been termed self-transcendent video content (Oliver et al., 2018). They found that a negative-to-positive emotional content shift results in more cognitive resources allocated to encoding (indicated by heart rate), a significant shift in the strength of positive emotional response (indicated by facial EMG), and an increase in prosocial intentions. Their experiment offers some support for the value of psychophysiological research as a methodological approach for experiments on emotional flow in health messages and provides a limited foundation for generating initial hypotheses. The experiment reported here builds on the work of Clayton et al. (2021) and examines whether emotional flow in vaccination messages produces heightened real-time psychophysiological engagement and whether such engagement corresponds with conscious emotional and attitudinal responses.

The dimensional theory of emotion has been proposed to be a more valid framework for the measurement of emotion in media processes and effects research grounded in media psychophysiology because psychophysiological measures more reliably index the valence and arousal dimensions of emotion than specific affective feelings (Bolls, 2010; Potter & Bolls, 2012). Emotional flow, as an emotional content shift, was manipulated in videos produced by the research team about the importance of COVID-19 vaccination and public health preventive actions as risk mitigation tactics. The produced videos either did or did not contain an emotional content shift in valence (positive or negative). This was done by writing copy focused on hope appeal, gain frame, and loss frame. The copy was used to create videos with and without an emotional content shift. Hope appeals have been theorized to increase the persuasiveness of a health message, especially when included with messages using fear appeal to communicate a health threat (Nabi & Myrick, 2019). Gain versus loss framing in message content is recognized as an important message feature in persuasion research. A recent systematic review reports that over half of the published studies on framing in health communication specifically focus on gain versus loss frames (Guenther et al., 2021). These content features—hope appeal, gain frame, and loss frame—were theorized to also be well-suited for the context of this study due to the ease of writing copy that strongly manipulates these specific features of messaging about COVID-19 vaccination and public health preventive actions. Consistent with the dimensional theory of emotion, loss framing was theorized to represent negative emotional valence, while gain framing and hope appeal were theorized to represent positive emotional valence. It is important to acknowledge that hope may not solely represent positive emotional valence. Hope, in previous health communication research, has been conceptualized as the desire for positive outcomes when dealing with adversity (Nabi & Prestin, 2016). Hope appeals in persuasive messages; however, it strongly presents emotionally positive outcomes.

A. Lang’s (2006, 2009) limited capacity model of motivated mediated message processing (LC4MP) provides a strong theoretical framework for research grounded in media psychophysiology like the present experiment, as well as hypotheses about how emotional flow might influence cognitive and emotional processing of a message. There are two highly relevant theoretical propositions in the LC4MP. One pertains to the notion of motivated attention—the proposition that cognition, emotion, and motivation are completely intertwined into our information processing system (P. J. Lang et al., 1997). The second is that information processing is fueled by activation of two biologically rooted motivational systems, the appetitive and the aversive systems. The appetitive system is generally activated by positive emotional valence, and the aversive system is generally activated by negative emotional valence. These motivational systems are independent, meaning either or both systems might become activated to varying degrees during exposure to a single health message. Emotional flow as emotional shifts, according to the LC4MP framework, could be conceptualized as a significant change in activation of the appetitive system compared to the aversive system or vice versa. Motivational system activation in either the appetitive or aversive system is conceptualized as variation in the arousal dimension of emotion.

No research grounded in media psychophysiology has directly investigated emotional flow in the context of health communication, but there is a small, related body of research on how variation in the emotional tone of content—conceptualized as variation in content activating the appetitive and aversive motivational systems—impacts cognitive and emotional processing of media content. The most relevant research investigates how content termed coactive, consisting of both positive and negative emotional content within a message, is processed (Keene & Lang, 2016; A. Lang et al., 2013; Leshner et al., 2018). This research generally indicates that content consisting of coactive—mixed positive and negative emotional tone—results in more cognitive resources allocated to encoding, increased arousal, and strong emotional responses that are consistent with variation in the valence of the emotional tone within a message.

Cognitive resources allocated to encoding are reliably indexed through heart rate deceleration during message exposure, arousal/motivational activation is indexed through skin conductance, and the valence of emotional responses is indexed through facial EMG (Potter & Bolls, 2012). Facial EMG recorded in this experiment only consisted of measurements of facial muscle activity in the corrugator muscle region located along the brow just above the eye socket. Activity in this muscle region is theorized to reliably reflect variation in the strength of negative emotional responding to a media message (Bolls et al., 2019; Tassinary & Cacioppo, 1992). The context (COVID-19 vaccination public health preventive policies) of this experiment, as well as the content of stimulus videos (a single announcer reading copy reflecting hope, gain framing, and loss framing), were theorized to primarily evoke variation in the strength of negative emotional responding during message exposure rather than strong, meaningful positive emotional responding. That is why the focus in this initial experiment was on negative emotional valence and recording of facial EMG activity in the corrugator muscle region. A strength of the media psychophysiology paradigm is the ability to investigate cognitive and emotional processes unfolding across time as an individual is exposed to a message (Bolls et al., 2019). This strength is realized by hypothesizing independent variables (e.g., emotional shifts) using time interactions in physiological data (Potter & Bolls, 2012).

Based on the above literature review, it is theorized that emotional flow, operationalized as the presence of an emotional shift in messaging about COVID-19 vaccination public health preventive actions, should increase cognitive resources allocated to encoding, arousal, and the strength of negative emotional responding. This will be evident in greater cardiac deceleration, skin conductance level change, and facial EMG activity in the corrugator muscle region across time for videos containing an emotional shift compared with videos that do not have an emotional shift in the content, which only contain positive emotion. This leads to the first set of hypotheses:

**H1.**  *There will be a significant Emotional Shift X Time interaction on heart rate change from baseline such that videos with an emotional shift will elicit greater cardiac deceleration across time compared to videos without an emotional shift.*

**H2.**  *There will be a significant Emotional Shift X Time interaction on skin conductance level change from baseline such that videos with an emotional shift will elicit higher levels of skin conductance across time compared to videos without an emotional shift.*

**H3.**  *There will be a significant Emotional Shift X Time interaction on corrugator facial EMG activity change from baseline such that videos with an emotional shift will elicit higher levels of corrugator facial EMG activity across time compared to videos without an emotional shift.*

### 2.2. Emotional Flow, Conscious Emotional Responses, and Persuasive Evaluation

In addition to investigating message features and their effects on cognitive and emotional processes indexed by psychophysiological measures, this study investigates conscious emotional and attitudinal responses, including feelings of hope and skepticism, and attitudes toward COVID-19 vaccine requirements. It is theoretically important to examine these outcomes together because emotional flow may influence persuasion not only by changing the emotions that individuals consciously experience but also by shaping how they allocate cognitive and emotional resources while processing a message.

Hope is a message-generated and future goal-oriented emotion that arises when individuals perceive the possibility of achieving a desirable future outcome (Chadwick, 2015; Lazarus, 1991, 1999; Nabi & Myrick, 2019). Drawing on the concept of emotional flow, emotions elicited by information presented early in a message are likely to shape emotional responses to information presented later (Nabi et al., 2018). The emotions-as-frames model suggests that emotions function as interpretive frames through which incoming stimuli are perceived, interpreted, and evaluated (Nabi, 2003, 2007). Once an emotion is elicited, the emotions-as-frames model predicts that emotion-congruent information becomes more accessible in memory, leading individuals to preferentially seek information that aligns with the emotions underlying motivational goals (Nabi et al., 2018). Applying this perspective to gain/loss framing, gain-framed messages highlight the potential for positive future outcomes, aligning closely with the central theme of hope (Nabi et al., 2018). Thus, this study argues that COVID-19 vaccine videos without emotional shift, manipulated by gain-framed messaging strategies and hope appeal, are more likely to elicit a greater feeling of hope compared to videos with emotional shift manipulated by loss-framed messaging strategies and hope appeal.

COVID-19 skepticism represents an important form of resistance to public health recommendations and policies. COVID-19 skepticism has been rampant (Levin & Bradshaw, 2022). COVID-19 skeptics do not believe in the effectiveness of preventive measures, such as mask-wearing, hand sanitization, and social distancing, and may perceive these measures as producing greater economic costs than benefits (Szántó & Dudás, 2022). COVID-19 skepticism can create challenges for implementing university vaccination requirements because individuals who question the effectiveness or necessity of COVID-19 preventive measures may be less receptive to messages advocating vaccination policies. Emotional appeals may provide one means of addressing such resistance. Prior research suggests that emotional shifts within messages can influence persuasive outcomes by changing the way individuals process subsequent information. In particular, Nabi and Green (2015) proposed that shifting emotional states may reduce defensive processing and thereby increase receptivity to persuasive messages. Thus, the emotional shift in COVID-19 vaccination messages could reduce skeptical responses by interrupting defensive reactions and encouraging audiences to consider the advocated policy more openly. To examine the influence of messages with emotional shifts on people’s feelings and attitudes toward university policies, we proposed:

**H4.**  *Participants will feel greater hope during exposure to videos without emotional shifts compared to videos with emotional shifts.*

**H5.**  *Participants will feel greater skepticism during exposure to videos without emotional shifts compared to videos with emotional shifts.*

**H6.**  *Participants will have stronger positive attitudes toward university policy about the COVID-19 vaccine requirement during exposure to videos with emotional shifts compared to videos without emotional shifts.*

## 3. Methods

### 3.1. Participants and Design

This study employed a two-level within-subject experimental design examining the effects of emotional shift. The emotional shift condition consisted of two levels: emotional shift and no emotional shift. Each condition was represented by two videos, resulting in four stimulus videos in total. All participants (N = 86) viewed four randomized videos in total, with the only difference being that the videos contained an emotional shift or no emotional shift. The two videos within each condition were treated as stimulus exemplars nested within that condition rather than as separate experimental conditions. Video presentation order was randomized across participants to balance potential order, fatigue, and carryover effects across the experimental conditions. The study protocol was approved by the university’s Institutional Review Board (#19070-002) prior to the start of recruitment. The study used flyers and emails to recruit a total of 86 participants (52 women, 33 men, and 1 transgender person) from different departments at a northwest university in the United States. Informed consent was provided before the start of the experiment. Each participant was compensated with a $20 grocery store gift card. Participants self-reported their race/ethnicity as White/Caucasian (n = 50, 58.1%), Black/African American (n = 2, 2.3%), Hispanic (n = 11, 12.8%), Asian (n = 13, 15.1%), Pacific Islander (n = 3, 3.5%), and as being more than two races/ethnicities (n = 7, 8.1%). Eighty-three participants reported having health insurance, and 3 participants reported not having health insurance. Data collection was in 2022 after COVID-19 vaccine eligibility had expanded to all U.S. adults in 2021 and our university began returning to full in-person operations and classes in Fall 2021.

### 3.2. Stimuli

Stimulus videos were scripted and produced by the research team for this experiment. This led to videos that had consistently controlled content and production features and that systematically varied on the independent variable of emotional shift. They were all roughly two minutes and thirty seconds, recorded with the same spokesperson—a young adult white male with professional broadcast experience—reading a script into a locked-down camera. Stimuli were purposefully produced to contain the same amount of information. Scripts did not vary significantly in length, and all scripts were focused on encouraging recommended prevention behaviors and aspects of college student life. The visual was simply a young adult male reading the script while looking at the camera.

The emotional shift was manipulated through the content of the scripts read by the narrator. Emotional shifts were created with a combination of a hope appeal and a loss framing (e.g., losing opportunities to bring back fun campus activities and life), which elicited an emotional shift between positive and negative emotional valence. Videos without emotional shifts only contained positive content; these videos were created by a combination of a hope appeal and a gain framing (e.g., benefits to completely bring a fun campus activity and life back). Each participant watched both videos, with and without an emotional shift, in a random order.

The message content feature manipulated was emotional shift (change in the emotional tone of the content). This was manipulated through the emotional tone of the script for each video. Distinct types of emotional tone were created through wording in the script. A hope script segment was written expressing the positivity of being able to return to enjoyable campus activities if prevention behaviors are adopted. This wording was completely toned to have a positive emotional valence. A negative emotional valence script segment was also written by emphasizing the loss (loss framing) of being able to enjoy fun campus activities if prevention behaviors are not adopted. The manipulation of emotional shift (change in the emotional tone of message content) was accomplished by editing the above segments of the scripts so that a message only contained a positive emotional tone created by the combination of message copy expressing hope and gain framing added to strengthen the hope appeal by emphasizing “regaining” the ability to enjoy fun campus activities. The concept of emotional flow is theoretically relevant to this experiment as a framework for understanding variation in human emotional experience during exposure to a message. However, the manipulation is strictly a manipulation of actual message content (copy) structured with words that reflect the emotional content, designed to produce messages that either had or did not have a shift (change) in the emotional tone of the content. The objective of this study was to provide practical insight into emotional content features under the direct control of message producers rather than advance theoretical understanding of emotional flow. The shift in the emotional content of the scripts for the messages used in this study is the content manipulation for this experiment that results in a change or no change in emotional flow of the content. The scripts for the stimuli can be found in Appendix A.

Pre-Test. A pre-test was conducted to determine what would be included as the in-person activities that young adults hope to experience again when they return to campus after COVID-19. Participants were asked to list ten university activities and to rank these activities by priority. The top three activities (e.g., sports games, concerts and movies on campus, and having a great time with friends) from the pre-test results were framed as desired activities when returning to campus as a hope appeal in the stimuli.

### 3.3. Procedure

Participants completed the experiment one at a time in a media psychophysiology lab. Data was collected through the iMotions 9.1 version platform (iMotions, Inc., Boston, MA, USA) using Shimmer hardware (Shimmer Research, Inc., Cambridge, MA, USA) to record physiological data. All procedures were in adherence to COVID-19 university and lab protocols. After obtaining informed consent, participants were prepped for the recording of physiological data (heart rate, skin conductance, and facial electromyography) following standard procedures (Potter & Bolls, 2012). A 30-s baseline was recorded during a black screen, and then participants viewed the four stimulus videos (emotional shift present/absent). Four stimulus videos were displayed in random order. Participants completed self-report measures after viewing each video, assessing the degree to which the participant felt hope and skepticism in response to the video and how the video impacted their attitudes toward university policies on vaccines. A 10-s instruction page (please sit relaxed and still. The video will be displayed) and a 30-s black screen were shown between viewing the videos in order to clear short-term memory of the first video viewed and limit carryover effects. Participants were then debriefed, compensated, thanked, and dismissed.

### 3.4. Dependent Variables

Cognitive Resources Allocated to Encoding is conceptualized as a specific form of human attention involving variation in effort focused on encoding sensory information (like video content) into short-term working memory (A. Lang, 2009). This dependent variable was measured by recording heart rate from an ECG waveform—a validated physiological indicator of cognitive resources allocated to encoding during exposure to video content (Potter & Bolls, 2012). The ECG recording was obtained utilizing a Lead I electrode placement consisting of two 8 mm Ag/AgCl gelled disposable surface electrodes placed on the forearms and a ground reference electrode on the left wrist. Heart rate was obtained from the inter-beat interval data (milliseconds between R spikes in the QRS waveform) and converted to bpm, averaging over 1-s intervals.

Arousal is conceptualized as activation of the embodied motivational systems (appetitive/aversive) involved in approach and avoidance responses as indicated by sympathetic nervous system activity. This conceptualization of arousal is theorized to reflect the intensity of emotional responses elicited by media content (A. Lang, 2009). Arousal was measured by recording skin conductance level, a validated physiological indicator of arousal that directly reflects variation in sympathetic nervous system activity (Potter & Bolls, 2012). Skin conductance was recorded by placing two reusable strap electrodes on the middle and ring fingers of the participant’s non-dominant hand.

Negative Emotional Response was conceptually defined as a specific response within the valence dimension of human emotion—as proposed in the dimensional theory of emotion (P. J. Lang et al., 1992). Strength of negative emotional response was recorded via facial electromyography (fEMG), specifically activity in the corrugator muscle region, which has been validated as an indicator of negative emotional response (Potter & Bolls, 2012). Activity within the corrugator muscle region was recorded by placing two 4 mm Ag/AgCI disposable electrodes above the inner corner of the eyebrow with a ground electrode placed on the bone of the forehead.

Hope emotion. Participants were asked to rate the extent to which they were likely to feel the emotion: hope as manifested in a feeling of “hope, optimism, and/or encouragement” on a 7-point scale ranging from “1 = not at all” to “7 = very much” for the no emotional shift video (*M* = 5.14, *SD* = 1.43) and emotional shift video (*M* = 4.29, *SD* = 1.70).

Skeptical emotion. Participants were asked to rate the extent to which they were likely to feel the emotion: skeptical, as manifested in a feeling of “cynic, skepticism, and/or distrust”, on a 7-point scale ranging from “1 = not at all” to “7 = very much” for the no emotional shift video (*M* = 2.13, *SD* = 1.33) and emotional shift video (*M* = 4.29, *SD* = 1.70). Skeptical emotion was measured as a proximal affective reaction directed toward the message. A higher score meant greater skeptical feeling toward the message.

Attitude toward COVID-19 policy for vaccine requirement. Participants were asked to indicate the level of agreement for the following statement: “I possess a positive attitude towards our university policy about the COVID-19 vaccine requirement” on a 7-point Likert scale ranging from “1 = strongly disagree” to “7 = strongly agree” for the video without emotional shifts (*M* = 5.86, *SD* = 1.62) and with emotional shifts (*M* = 5.62, *SD* = 1.83). Policy attitude was measured as the distal evaluation of the advocated university COVID-19 vaccine requirement. A higher score meant stronger agreement on a positive attitude toward that policy.

## 4. Results

This study employed a psychophysiology experimental design to investigate the mental process as well as emotional and attitudinal responses to messages with emotional shifts regarding COVID-19 public health preventive actions.

### 4.1. Psychophysiology Data Pre-Processing

The four stimulus videos ranged in duration from two minutes 24 s (144 s) to two minutes 38 s (158 s). Psychophysiological activity was recorded continuously throughout each video. To standardize the number of observations across videos of slightly different durations while retaining information about patterns of physiological change over time, each video recording was divided into 36 equal-progression intervals. Consequently, interval duration varied from 4 s to 4.39 s across videos. Values for heart rate, skin conductance, and facial EMG were averaged within each interval, yielding 36 observations per physiological measure for each participant-video combination. These intervals represented comparable relative positions in the progression of each video rather than identical periods of absolute time. The heart-rate analysis therefore focuses on the trajectory of cardiac activity across the full 144–158 s video. Individual four-second intervals were not interpreted as independent tonic responses. For each physiological measure, a baseline value was calculated by averaging activity during the 30-s pre-exposure baseline period. Baseline-corrected change scores were computed by subtracting the corresponding baseline mean from each of the 36 interval values. The confirmatory nature of the analysis was conducted by running a series of repeated measures ANOVA in SPSS 28 to test the hypotheses.

### 4.2. Attention

Attention was indexed by measuring participants’ heart rate. Equipment failures occurred with nine participants, so heart-rate analyses were collected from 77 participants. In terms of the nature of emotional shifts, this study focused on tonic responses to examine how people’s mental processing occurred over comparatively long periods of time.

**Figure 1:**
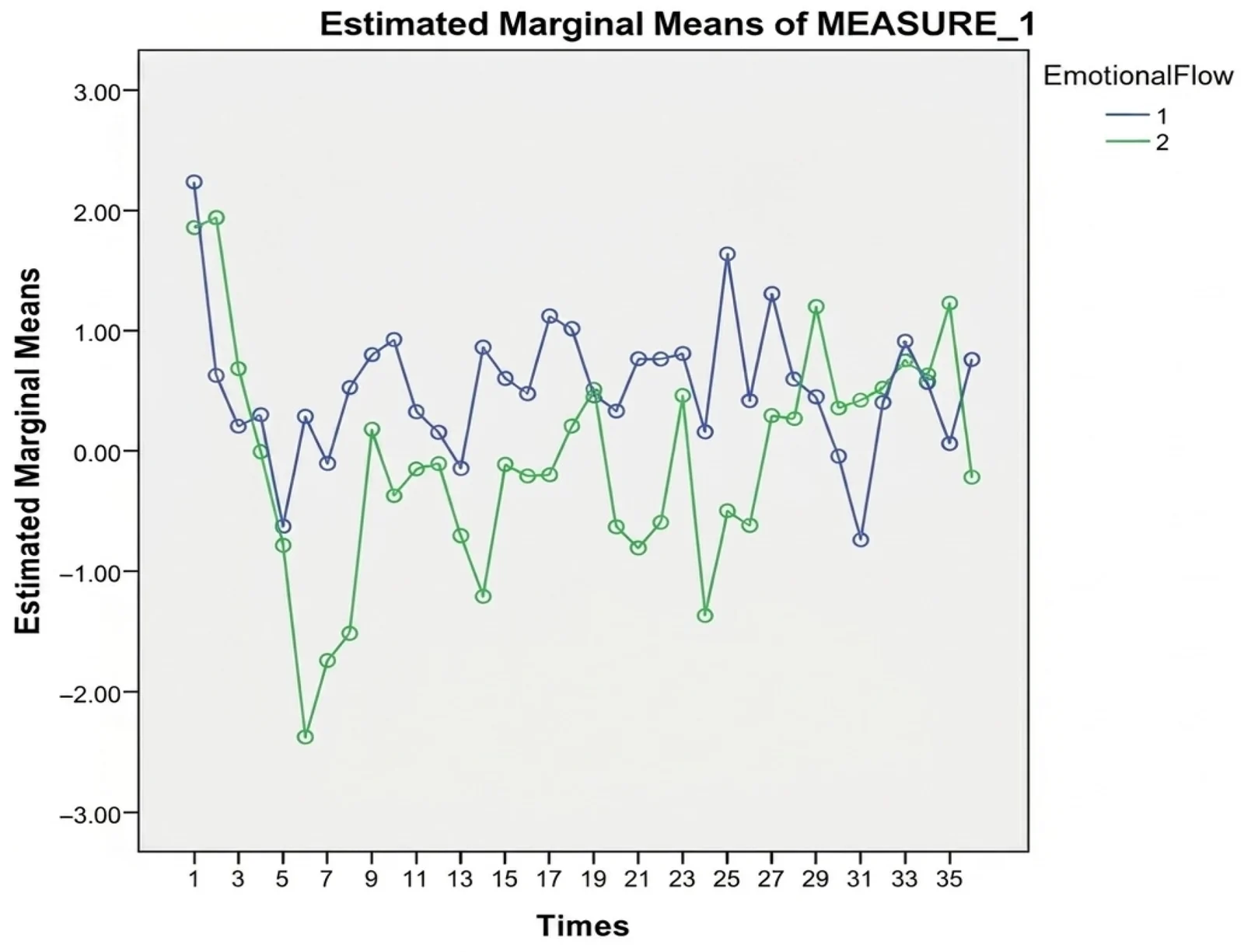
Emotional flow in heart rate changes across time. **Notes:** Blue line (emotional flow 1) represented as “no emotional shifts”, meaning the videos remain positive emotions only; Green line (emotional flow 2) represented as “emotional shifts” from negative to positive emotional changes through the videos.

**H1** predicted ***greater heart rate deceleration over time*** for participants who watched the emotional-shift videos than for those who watched the no-emotional-shift videos. A significant two-way interaction of emotional flow and time was found, *F*(19.32, 1448.60) = 1.67, *p* = 0.034, *ŋ*^2^ = 0.022. As Figure 1 shows, exposure to videos containing emotional shifts evoked greater heart rate deceleration over time compared to videos with no emotional shifts. H1 was supported.

### 4.3. Arousal

**Figure 2:**
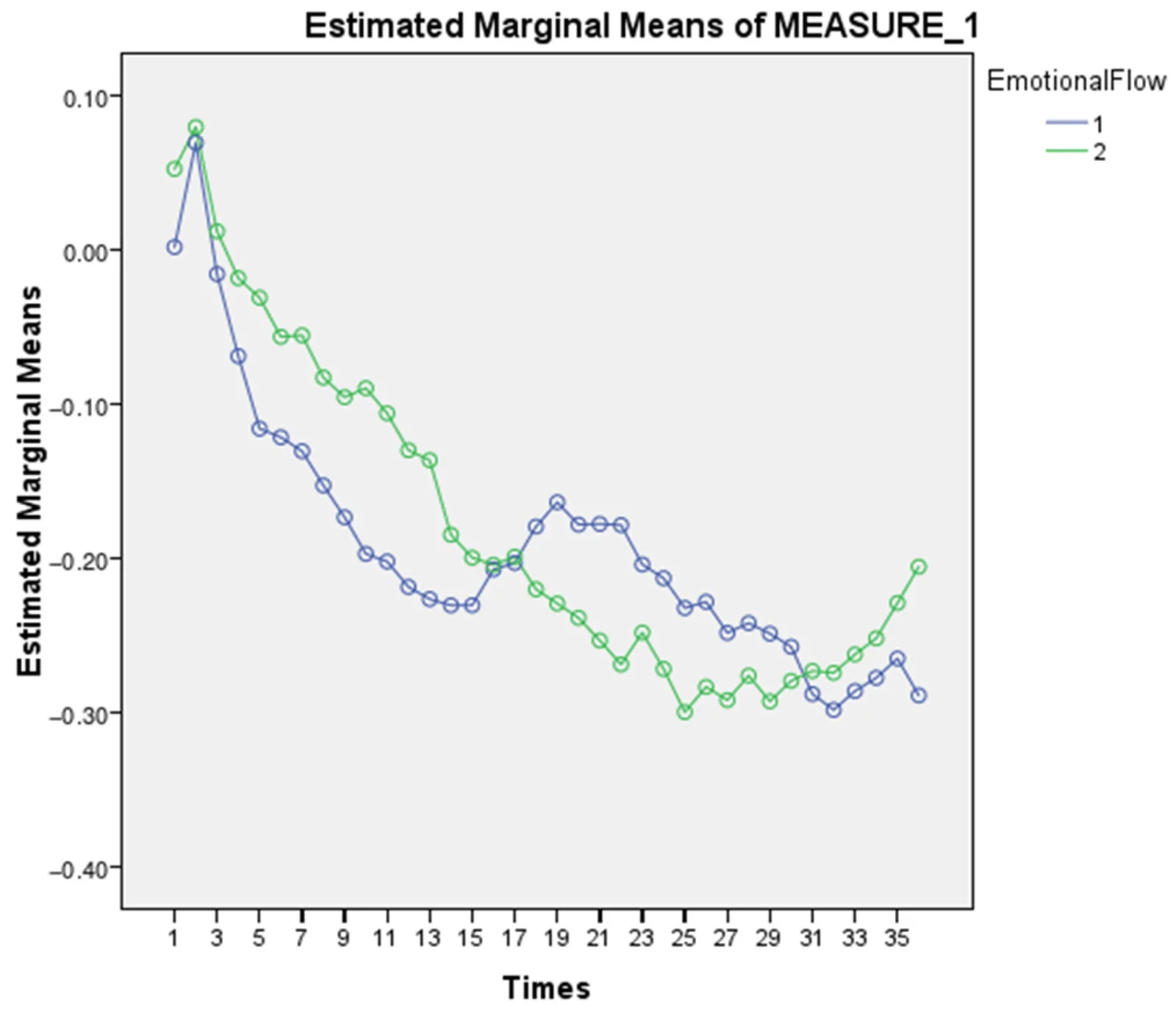
Emotional flow in arousal from skin conductance across time. **Notes:** Blue line (emotional flow 1) represented as “no emotional shifts”, meaning the videos remain positive emotions only; Green line (emotional flow 2) represented as “emotional shifts” from negative to positive emotional changes through the videos.

Arousal was indexed by measuring participants’ skin conductance. **H2** predicted ***greater arousal over time*** for participants who watched the emotional shift videos than for those who watched the no emotional shift videos. A significant two-way interaction of emotional shift and time was found, *F*(3.95, 280.60) = 2.41, *p* = 0.050, *ŋ*^2^ = 0.033. As Figure 2 shows, exposure to videos containing emotional shifts evoked greater arousal over time compared to videos with no emotional shifts. H2 was supported.

### 4.4. Negative Emotional Response

**Figure 3:**
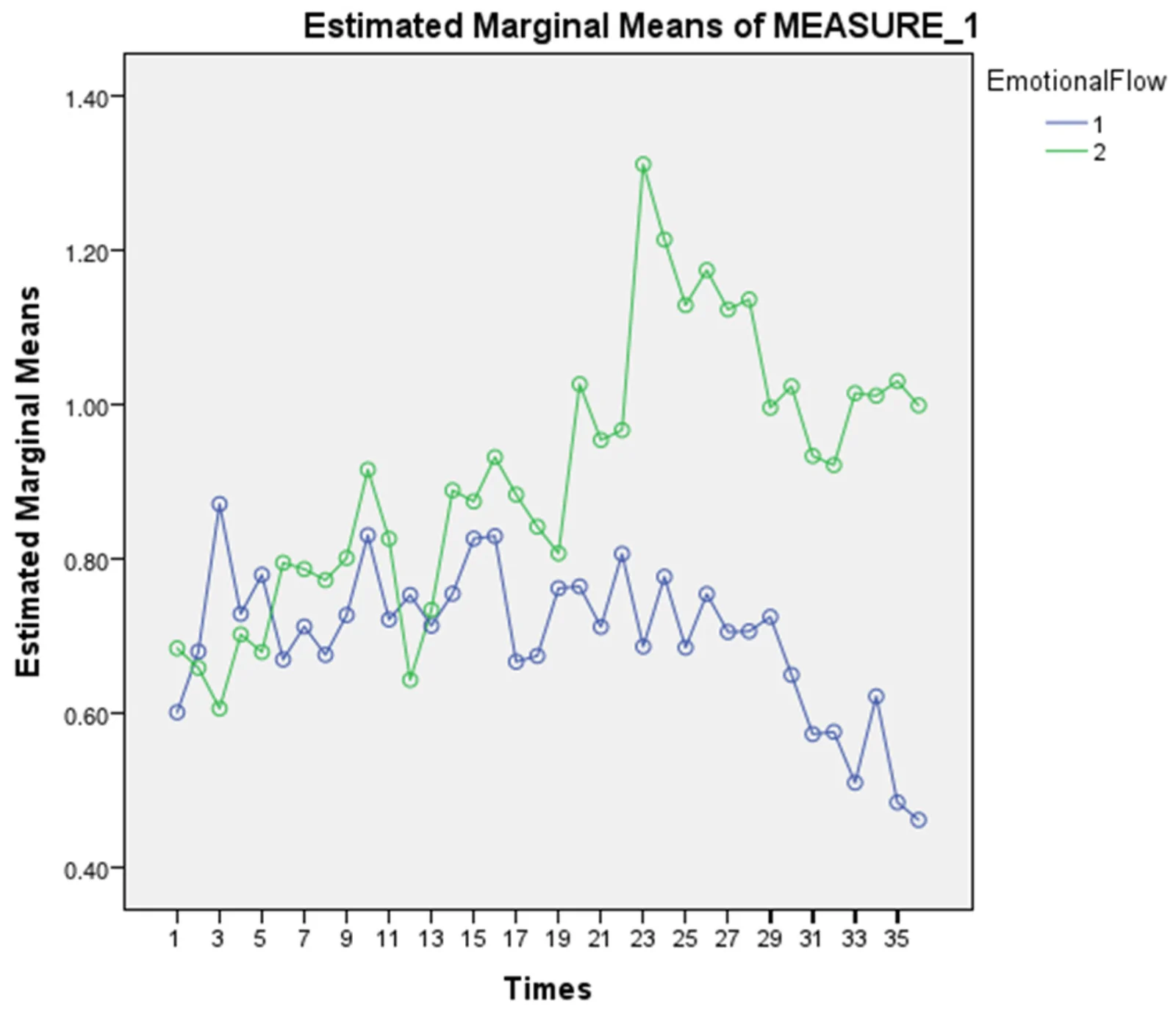
Emotional flow in corrugator supercilii muscle activities across time. **Notes:** Blue line (emotional flow 1) represented as “no emotional shifts”, meaning the videos remain positive emotions only; green line (emotional flow 2) represented as “emotional shifts” from negative to positive emotional changes through the videos.

The negative emotional response was indexed by measuring participants’ corrugator muscle activity. Equipment failures occurred with 33 participants, so the fEMG analysis was based on 53 participants. **H3** predicted ***greater negative emotional responses over time*** for participants who watched the emotional shift videos than for those who watched the no emotional shift videos. A significant two-way interaction of emotional shift and time was found, *F*(8.80, 449.02) = 2.98, *p* = 0.002, *ŋ*^2^ = 0.055. As Figure 3 shows, exposure to videos containing emotional shifts evoked a greater negative emotional response over time compared to videos with no emotional shifts. H3 was supported.

### 4.5. Self-Reported Emotional and Attitudinal Responses

**H4** predicted ***a greater feeling of hope*** for participants who watched the no-emotional shift videos than for those who watched the emotional shift videos. A significant main effect was found, *F*(1, 82) = 18.22, *p* ≤ 0.000, *ŋ*^2^ = 0.182. People had a greater feeling of hope when watching videos with no emotional shifts (*M* = 5.12, *SE* = 0.155) than with emotional shifts (*M* = 4.30, *SE* = 0.181). H4 was supported.

**H5** predicted ***a greater feeling of skepticism*** for participants who watched the no-emotional shift videos than for those who watched the emotional shift videos. Results showed a significant main effect, *F*(1, 82) = 13.84, *p* ≤ 0.000, *ŋ*^2^ = 0.144. However, people had a greater feeling of skepticism when watching videos with emotional shifts (*M* = 2.82, *SE* = 0.173) than with no emotional shifts (*M* = 2.15, *SE* = 0.145). H5 was not supported.

**H6** predicted ***a greater positive attitude toward university policy about the COVID-19 vaccine requirement*** for participants who watched the emotional shift videos than for those who watched the no emotional shift videos. Results showed a significant main effect, *F*(1, 82) = 6.17, *p* = 0.015, *ŋ*^2^ = 0.070. However, people had a greater positive attitude toward university policy about receiving the COVID-19 vaccine requirement when watching videos with no emotional shifts (*M* = 5.85, *SE* = 0.174) than with emotional shifts (*M* = 5.63, *SE* = 0.195). H6 was not supported.

## 5. Discussions

The research objective for this study was to investigate how emotional flow in health crises and risk messaging—like COVID-19—impacts students’ mental processes and attitudes. Results indicate that emotional shifts lead to an increase in cognitive resources allocated to encoding the message, higher levels of sympathetic arousal, and stronger negative emotional responses. In addition, emotional shifts resulted in less feeling of hope and positive attitudes toward the policy of the COVID-19 vaccine mandate, but greater skepticism.

First, the results from the effects of emotional shifts on psychophysiological processes (e.g., high negative emotional response, intensity of arousal, and attention) provide evidence of how the human brain dynamically interacts with different emotional features in health crisis and risk messages regarding the Media Psychophysiology paradigm (Bolls et al., 2019). The results also support the idea that changes in emotional experiences evoked by processing emotional content influence the allocation of cognitive resources for attention to and the intensity of emotional engagement with message elements about COVID-19 vaccination (Nabi & Green, 2015). In addition, the findings indicate emotional flow as a dynamic process that increases the psychological significance of the COVID-19 vaccine messages. Compared to static positive or negative emotions, emotional change plays a more significant role in capturing attention by signaling to the human brain that something important has happened in the media environment during COVID-19 message exposure. The results also provide evidence that emotion and cognition (e.g., attention) are closely intertwined (Brosch et al., 2013), indicating that emotionally significant information in COVID-19 messages is prioritized to be received with limited cognitive resources.

In addition, the findings about the effect of emotional flow on greater attentional processing and arousal during COVID-19 message exposure align with LC4MP (A. Lang, 2006, 2009), which posits that motivationally and emotionally relevant messages elicit automatic resource allocation and activate human motivational and cognitive systems. Thus, emotional messages about the COVID-19 vaccine may influence where viewers allocate their cognitive attention and how they process emotionally salient message content. The emotional change elicited by noticeable emotional shifts can increase the message’s motivational significance, which prompts viewers to allocate more cognitive resources to understand what happened in the message content.

Second, one of the interesting findings was that emotional flow messages about the COVID-19 vaccine elicited a greater feeling of skepticism compared to no emotional flow messages, which is contradictory to the concept of emotional flow that reduces defensive processing and the feeling of skepticism (Nabi & Green, 2015). Regarding the emotions-as-frames perspective (Nabi, 2003, 2007; Nabi et al., 2018), emotions not only arise from message exposure but also shape how individuals interpret, evaluate, and respond to the message. Thus, an emotional shift serves as an emotional cue to make the persuasive intent of the message about the COVID-19 vaccine more salient, prompting individuals to be more critical in evaluating message claims about the vaccine’s effectiveness and expectations for future life, which in turn leads to greater skepticism.

Third, about the effects of emotional flow on reducing positive attitudes toward the policy of COVID-19 vaccine requirement at the university, the finding suggests that the persuasive effects of emotional flow might depend on the types of outcomes being evaluated, even though emotional shifts can increase emotional engagement and attention. Regarding emotion-as-frames (Nabi, 2003, 2007; Nabi et al., 2018), emotional flow messages introduce changing affective cues, which could activate participants to evaluate the COVID-19 vaccine policy and considerations differently during COVID-19 message exposure, while messages without emotional flow may provide a more stable emotional frame, which allows participants to evaluate the vaccine policy with more consistent evaluation and considerations. An emotionally stable and cognitively coherent basis for evaluating the university’s COVID-19 vaccination requirement increases positive attitudes toward the COVID-19 vaccine policy when viewing messages without emotional shifts. Moreover, the affect-as-information theory (Schwarz & Clore, 1983; Schwarz, 2012) posits that people may attend to their momentary feelings as a source of information to form judgments and assumes that feelings influence people’s perceived information value and information processing style. Thus, the absence of changing emotional cues in messages without emotional flow might reduce emotional complexity, minimize perceptions of persuasive intent, and allow participants to focus more directly on the informational and institutional considerations of the COVID-19 requirement, which could increase their positive attitudes toward that policy.

In addition, regarding the psychological reactance theory (Brehm, 1989), the motivational state, known as psychological reactance, motivates individuals to resist the restriction and attempt to restore the threatened freedom when they perceive that their behavioral freedom is threatened or restricted, which causes their motivational arousal. Because the university vaccination requirement could be perceived as constraining individual choice, messages with emotional flow could introduce competing affective cues to heighten that perception, persuasive intent, or external control, which might activate participants’ psychological reactance and lead them to resist the policy support, even though they have greater emotional engagement.

### 5.1. Theoretical Implications

First, results from this study indicate that emotional flow can intensify message processing without improving persuasion and may, under some conditions, increase skepticism and weaken favorable policy attitudes. Regarding the emotional flow on psychophysiological processing of messages about the COVID-19 vaccine, from the LC4MP perspective, the findings advance the emotional flow (Nabi & Green, 2015) in vaccine communication by investigating the crucial roles of unconscious cognitive and emotional processes in health messages with emotional appeals. This study, based on the results of cognitive and emotional processes in real-time, also answers the fundamental question of how the human brain dynamically interacts with different emotional features in health content across time, influencing the encoding of messages, attention, and emotional arousal, which fills the gaps in existing emotional flow studies.

In addition, the results from the emotional flow on psychological processing and persuasion outcomes also indicate that greater emotional engagement can coexist with weaker persuasion because the emotional experience formed by emotional shifts serves as a signal to impact how people interpret and evaluate messages that are manipulative and threatening, regarding affect-as-information theory (Schwarz & Clore, 1983; Schwarz, 2012). Also, regarding psychological reactance theory (Brehm, 1989), if emotional flow makes the messages feel more emotionally forceful or controlling, individuals could have psychological reactance, increased resistance, and have less favorable policy attitudes when they perceive emotional force or control as a threat to their behavioral freedom. This study advances emotional flow studies by showing that emotional flow may have downstream consequences for cognitive evaluation and policy attitudes. Emotional flow, increasing emotional processing, does not necessarily translate into persuasion. Instead, emotional flow may heighten attention and engagement in ways that encourage critical evaluation, skepticism, and resistance to a health policy, such as vaccination. The findings of this study theoretically contribute to the emotional flow literature that emotional flow may amplify processing without determining the valence of the resulting judgment.

Second, this study examines the effects of emotional shifts through hope appeals and gain-loss framing. The results support the claim that inducing positive or negative emotions can enhance the influence of gain-loss frames (Nabi et al., 2020). This study also extends gain-loss framing and hope appeal by integrating emotional dynamics, which proves that gain-loss framing effects are not solely determined by the valence of message information but may also depend on the emotional trajectory established before or during exposure to the frame. Vaccination messages with emotional shifts by framing the positive and negative consequences and future expectations of hope appeals can generate different levels of emotional involvement and excitation transfer processes. However, the result about the emotional flow on less favorable attitudes toward the vaccine policy indicates that emotion enhances the psychological impact of gain-loss framing, but the direction of the attitude depends on how individuals interpret and evaluate the framed information.

Further consideration of the psychophysiological results is warranted. The advantage of psychophysiological measures is to index variation in cognitive and emotional responses in real time during exposure to the message. Figure 1 (heart rate) and Figure 3 (corrugator facial EMG) illustrate this advantage as well as some nuances in how the messages used in this experiment were processed. Despite beginning with content that is similarly emotionally toned, messages with and without an emotional shift are processed somewhat differently across the whole message. This may be because messages with an emotional shift differ qualitatively from messages without an emotional shift, even before the shift occurs within the message. In real-world media production, this is the case. The vocal tone and facial expressions of an effective spokesperson are going to be different from the start of a message for the two different message strategies tested in this experiment. This will lead to similarly toned messages at the beginning of the message that are actually different in terms of message strategy to be processed differently.

### 5.2. Limitations and Future Directions

There are some limitations. First, the participants were recruited from one university in the United States, so the findings may not generalize to other universities in other countries. Future studies should conduct research across different universities and countries to enable generalization. Second, this study only focused on one vaccine mandate (e.g., the COVID-19 vaccine). Future studies should explore various contexts related to vaccine mandates, including those concerning other health concerns and different vaccines. Third, this study was unable to collect positive emotions using facial electromyography, so future studies should collect positive and negative emotions using facial electromyography for emotional shifts with different emotional valences. Also, future studies should more carefully examine the wide range of pre-existing attitudes and systematically investigate the top-down effects on how individuals process and respond to messages during public health crises. Fourth, this study did not measure vaccine hesitancy, anti-vaccine attitudes, and actual vaccination behavior about the COVID-19 vaccine. Thus, the present findings were not evidence that emotional flow increased or decreased vaccine hesitancy. Rather, the findings indicate differences between message conditions in skeptical emotional responses and policy attitudes. However, vaccine hesitancy and anti-vaccine attitudes are barriers to vaccine acceptance (Chou & Budenz, 2020; Nan et al., 2022), so future studies should measure these variables between pre-and-post tests. Also, future studies should conduct longitudinal research to evaluate the vaccine campaigns on actual vaccination behavior. Fifth, the attitude toward the COVID-19 vaccine requirement policy was measured using a single item, so the measure may not fully represent the attitude construct and internal consistency. Future research should employ validated multi-item measures for attitudes toward vaccination policies. Nevertheless, we believe the present results offer actionable insights for university communication administrators and researchers interested in using psychophysiological measures to assess health-related behavioral outcomes, such as vaccinations.

## Data Availability

The data that support the findings of this study are not publicly available due to privacy and ethical restrictions but are available from the corresponding author upon reasonable request.

## Appendix A

### Appendix A.1. Content for No Emotional Shift

**[Hope placement]** If you are like me, you have really been hoping for the time when we can all truly relax and enjoy all of the great experiences that make living in a university community so much fun! The experiences we have all had over the past year as a result of how much this virus spread and the severe sickness it can cause prove that despite progress in reducing the spread of COVID-19, the future is extremely uncertain. The likelihood that the spread and severity of COVID-19 can be consistently controlled and reduced to the point that we can once again return to a much more enjoyable daily life is unknown but also highly depends on our actions. Widespread vaccination offers hope for a more enjoyable life. If all of us who can medically and morally receive the COVID-19 vaccine get vaccinated as soon as possible, we can have hope and spread hope for a much better return to enjoying all the things we have missed. **[Gain framing]** Getting the COVID-19 vaccine can reduce your chance of getting COVID-19. If you receive the COVID-19 vaccine, you can also protect your loved ones and WSU communities’ healthy and safety. More importantly, getting the COVID-19 vaccine can bring Cougar life back. You can watch football games with your friends and families in Martin Stadium. Before and after football games, you can go to Ferdinand’s Ice Cream Shoppe and eat delicious and special WSU ice cream with your friends and families. Also, you can and will create countless memorable, personal, and fun times with your classmates and friends at WSU campus. Go get the COVID-19 vaccine; you can experience and enjoy the wonderful and special Cougar life soon!

**[Gain framing]** Getting the COVID-19 vaccine can reduce your chance of getting COVID-19. If you receive the COVID-19 vaccine, you can also protect your loved ones and WSU communities’ healthy and safety. More importantly, getting the COVID-19 vaccine can bring Cougar life back. You can watch football games with your friends and families in Martin Stadium. Before and after football games, you can go to Ferdinand’s Ice Cream Shoppe and eat delicious and special WSU ice cream with your friends and families. Also, you can and will create countless memorable, personal, and fun times with your classmates and friends at WSU campus. Go get the COVID-19 vaccine; you can experience and enjoy the wonderful and special Cougar life soon! **[Hope placement]** If you are like me, you have really been hoping for the time when we can all truly relax and enjoy all of the great experiences that make living in a university community so much fun! The experiences we have all had over the past year as a result of how much this virus spread and the severe sickness it can cause prove that despite progress in reducing the spread of COVID-19, the future is extremely uncertain. The likelihood that the spread and severity of COVID-19 can be consistently controlled and reduced to the point that we can once again return to a much more enjoyable daily life is unknown but also highly depends on our actions. Widespread vaccination offers hope for a more enjoyable life. If all of us who can medically and morally receive the COVID-19 vaccine get vaccinated as soon as possible, we can have hope and spread hope for a much better return to enjoying all the things we have missed.

### Appendix A.2. Content for Emotional Shifts

**[Hope placement]** If you are like me, you have really been hoping for the time when we can all truly relax and enjoy all of the great experiences that make living in a university community so much fun! The experiences we have all had over the past year as a result of how much this virus spread and the severe sickness it can cause prove that despite progress in reducing the spread of COVID-19, the future is extremely uncertain. The likelihood that the spread and severity of COVID-19 can be consistently controlled and reduced to the point that we can once again return to a much more enjoyable daily life is unknown but also highly depends on our actions. Widespread vaccination offers hope for a more enjoyable life. If all of us who can medically and morally receive the COVID-19 vaccine get vaccinated as soon as possible, we can have hope and spread hope for a much better return to enjoying all the things we have missed. **[Loss framing]** Not getting the COVID-19 vaccine can increase your chance of getting COVID-19. If you do not receive the COVID-19 vaccine, your loved ones and WSU communities can be threatened by you. More importantly, without getting the COVID-19 vaccine, you rarely can bring the Cougar life back. You cannot watch football games with your friends and families in Martin Stadium. Before and after football games, you cannot go to Ferdinand’s Ice Cream Shoppe and cannot eat delicious and special WSU ice cream with your friends and families. Also, you can only see your classmates and friends through Zoom, so you lose the opportunity to create countless memorable, personal, and fun times with your classmates and friends at WSU campus. If you do not get the COVID-19 vaccine, you can never experience and enjoy the wonderful and special Cougar life soon!

**[Loss framing]** Not getting the COVID-19 vaccine can increase your chance of getting COVID-19. If you do not receive the COVID-19 vaccine, your loved ones and WSU communities can be threatened by you. More importantly, without getting the COVID-19 vaccine, you rarely can bring the Cougar life back. You cannot watch football games with your friends and families in Martin Stadium. Before and after football games, you cannot go to Ferdinand’s Ice Cream Shoppe and cannot eat delicious and special WSU ice cream with your friends and families. Also, you can only see your classmates and friends through Zoom, so you lose the opportunity to create countless memorable, personal, and fun times with your classmates and friends at WSU campus. If you do not get the COVID-19 vaccine, you can never experience and enjoy the wonderful and special Cougar life soon! **[Hope placement]** If you are like me, you have really been hoping for the time when we can all truly relax and enjoy all of the great experiences that make living in a university community so much fun! The experiences we have all had over the past year as a result of how much this virus spread and the severe sickness it can cause prove that despite progress in reducing the spread of COVID-19, the future is extremely uncertain. The likelihood that the spread and severity of COVID-19 can be consistently controlled and reduced to the point that we can once again return to a much more enjoyable daily life is unknown but also highly depends on our actions. Widespread vaccination offers hope for a more enjoyable life. If all of us who can medically and morally receive the COVID-19 vaccine get vaccinated as soon as possible, we can have hope and spread hope for a much better return to enjoying all the things we have missed.
